# Supplementary material for: Caught in a trap: DNA contamination in tsetse xenomonitoring can lead to over-estimates of Trypanosoma brucei infection
Source: PLoS Negl Trop Dis. 2024 Aug 12;18(8):e0012095. doi: 10.1371/journal.pntd.0012095 (PMC11341098; doi:10.1371/journal.pntd.0012095)
Supplement: S1 Table — (PDF) [file pntd.0012095.s001.pdf]

Treatment	Replicate	Infected			Uninfected		
		M	F	Total IF	M	F	Total UF
**T1**	**A**	1	8	9	2	1	3
	**B**	4	5	9	2	1	3
	**C**	4	5	9	2	1	3
**T2**	**A**	4	2	6	2	4	6
	**B**	4	2	6	2	4	6
	**C**	4	2	6	2	4	6
**T3**	**A**	1	0	1	5	6	11
	**B**	1	0	1	5	6	11
	**C**	1	0	1	5	6	11
**C0**	**A**	0	0	0	5	7	12
	**B**	0	0	0	6	6	12
	**C**	0	0	0	6	6	12

S1 Table: A table displaying *G. m. morsitans* sex and infection ratios for trap cage experiments. M = male, F = female, IF = infected fly, UF = naïve uninfected fly.
