# Supplementary material for: Caught in a trap: DNA contamination in tsetse xenomonitoring can lead to over-estimates of Trypanosoma brucei infection
Source: PLoS Negl Trop Dis. 2024 Aug 12;18(8):e0012095. doi: 10.1371/journal.pntd.0012095 (PMC11341098; doi:10.1371/journal.pntd.0012095)
Supplement: S2 Table — Tarangire NP = Tarangire National Park. (PDF) [file pntd.0012095.s002.pdf]

Trap ID	Transect	Region	Latitude	Longitude
TA-1	TA	Tarangire NP	157791.8	-432982
TA-2	TA	Tarangire NP	157758.3	-433093
TA-3	TA	Tarangire NP	160220.6	-433927
TA-4	TA	Tarangire NP	160390.8	-434067
TA-5	TA	Tarangire NP	162344	-435548
TA-6	TA	Tarangire NP	162421.2	-435634
TA-7	TA	Tarangire NP	166310.1	-439209
TA-8	TA	Tarangire NP	166425.8	-439176
TA-9	TA	Tarangire NP	169880.1	-440627
TA-10	TA	Tarangire NP	169987.6	-440676
TA-11	TA	Tarangire NP	175093	-440977
TA-12	TA	Tarangire NP	175332.8	-441007
TA-13	TA	Tarangire NP	177523	-444401
TA-14	TA	Tarangire NP	177595.1	-444307
TA-15	TA	Tarangire NP	180261.1	-447310
TA-16	TA	Tarangire NP	180260.7	-446981
TB-1	TB	Tarangire NP	183902.2	-453155
TB-2	TB	Tarangire NP	183768.1	-453218
TB-3	TB	Tarangire NP	182148.9	-452704
TB-4	TB	Tarangire NP	182112.8	-452564
TB-5	TB	Tarangire NP	179855.6	-452141
TB-6	TB	Tarangire NP	179708.8	-452127
TB-7	TB	Tarangire NP	177981.8	-450880
TB-8	TB	Tarangire NP	177902.2	-450792
TB-9	TB	Tarangire NP	176262.9	-449731
TB-10	TB	Tarangire NP	176173.9	-449653
TB-11	TB	Tarangire NP	176600	-446489
TB-12	TB	Tarangire NP	176705.1	-446403
TB-13	TB	Tarangire NP	174817.8	-444388
TB-14	TB	Tarangire NP	174923.5	-444350
TB-15	TB	Tarangire NP	177013.3	-438641
TB-16	TB	Tarangire NP	177137.6	-438599
TB-17	TB	Tarangire NP	175713.3	-436707
TB-18	TB	Tarangire NP	175797.3	-433914
TB-19	TB	Tarangire NP	175752.6	-433802
TC-1	TC	Tarangire NP	173251	-421652
TC-2	TC	Tarangire NP	173220.6	-421768
TC-3	TC	Tarangire NP	174119.1	-424909
TC-4	TC	Tarangire NP	174101.6	-425026
TC-5	TC	Tarangire NP	174093.3	-427756
TC-6	TC	Tarangire NP	174194	-427822
TC-7	TC	Tarangire NP	176538.1	-429781
TC-8	TC	Tarangire NP	176646.3	-429786
TC-9	TC	Tarangire NP	179625.5	-429866
TC-10	TC	Tarangire NP	179737.1	-429824
TC-11	TC	Tarangire NP	181196.1	-430670
TC-12	TC	Tarangire NP	181276.2	-430743
TC-13	TC	Tarangire NP	185134.2	-432707
TC-14	TC	Tarangire NP	185208.6	-432647
TC-15	TC	Tarangire NP	185478.8	-430603
TC-16	TC	Tarangire NP	185353.4	-430546
BA-1	BA	Lobosoiret	196443.2	-474089
BA-2	BA	Lobosoiret	196540.9	-474048
BA-3	BA	Lobosoiret	198862.3	-475777
BA-4	BA	Lobosoiret	198961.9	-475856
BA-5	BA	Lobosoiret	201891.1	-476718
BA-6	BA	Lobosoiret	201973.1	-476656
BA-7	BA	Lobosoiret	204397.6	-476615
BA-8	BA	Lobosoiret	204495.8	-476564
BA-9	BA	Lobosoiret	206114.6	-475525
BA-10	BA	Lobosoiret	206145.5	-475429
BA-11	BA	Lobosoiret	201296	-496248
BA-12	BA	Lobosoiret	201393.9	-496203
BA-13	BA	Lobosoiret	204968	-492717
BA-14	BA	Lobosoiret	205045.9	-492630
BA-15	BA	Lobosoiret	206585.7	-489703
BA-16	BA	Lobosoiret	206612.1	-489587
BA-17	BA	Lobosoiret	207077	-487013
BA-18	BA	Lobosoiret	207040.6	-486886
BA-19	BA	Lobosoiret	207231.9	-482912
BA-20	BA	Lobosoiret	207254.5	-482776
BB-1	BB	Kimotorok	215096.5	-441900
BB-2	BB	Kimotorok	215074.3	-442017
BB-3	BB	Kimotorok	214548.5	-446742
BB-4	BB	Kimotorok	214556.6	-446849
BB-5	BB	Kimotorok	215311.5	-451567
BB-6	BB	Kimotorok	215371.5	-451687
BB-7	BB	Kimotorok	216877	-455466
BB-8	BB	Kimotorok	216897	-455571
BB-9	BB	Kimotorok	218574.7	-459906
BB-10	BB	Kimotorok	218591.7	-460021
BB-11	BB	Kimotorok	219323.5	-469269
BB-12	BB	Kimotorok	219236.6	-469328
BB-13	BB	Kimotorok	215354	-470747
BB-14	BB	Kimotorok	215238.1	-470780
BB-15	BB	Kimotorok	209051.2	-474730
BB-16	BB	Kimotorok	209059.9	-474853
BB-17	BB	Kimotorok	208837.6	-478110
BB-18	BB	Kimotorok	208833.1	-478251

S2 Table: A table displaying transect, region and coordinates for each Nzi trap set as part of the study. Tarangire NP = Tarangire National Park.
