# Supplementary material for: Caught in a trap: DNA contamination in tsetse xenomonitoring can lead to over-estimates of Trypanosoma brucei infection
Source: PLoS Negl Trop Dis. 2024 Aug 12;18(8):e0012095. doi: 10.1371/journal.pntd.0012095 (PMC11341098; doi:10.1371/journal.pntd.0012095)
Supplement: S1 Fig — (a) and (b) show tsetse being blood-fed in solitary cells; (c) tsetse resting after bloodmeal; (d) tsetse solitary cells suspended above filter paper discs in rack; (e) collection of tsetse faecal samples on filter paper; (f) an infected tsetse marked with green oil paint; (g) experiment trap cages; (h) two infected tsetse (fly IDs 87 and 109) copulating inside trap cage during experiment; (i) dissected tsetse midgut infected with T. brucei as viewed under a microscope (400X). (PDF) [file pntd.0012095.s003.pdf]

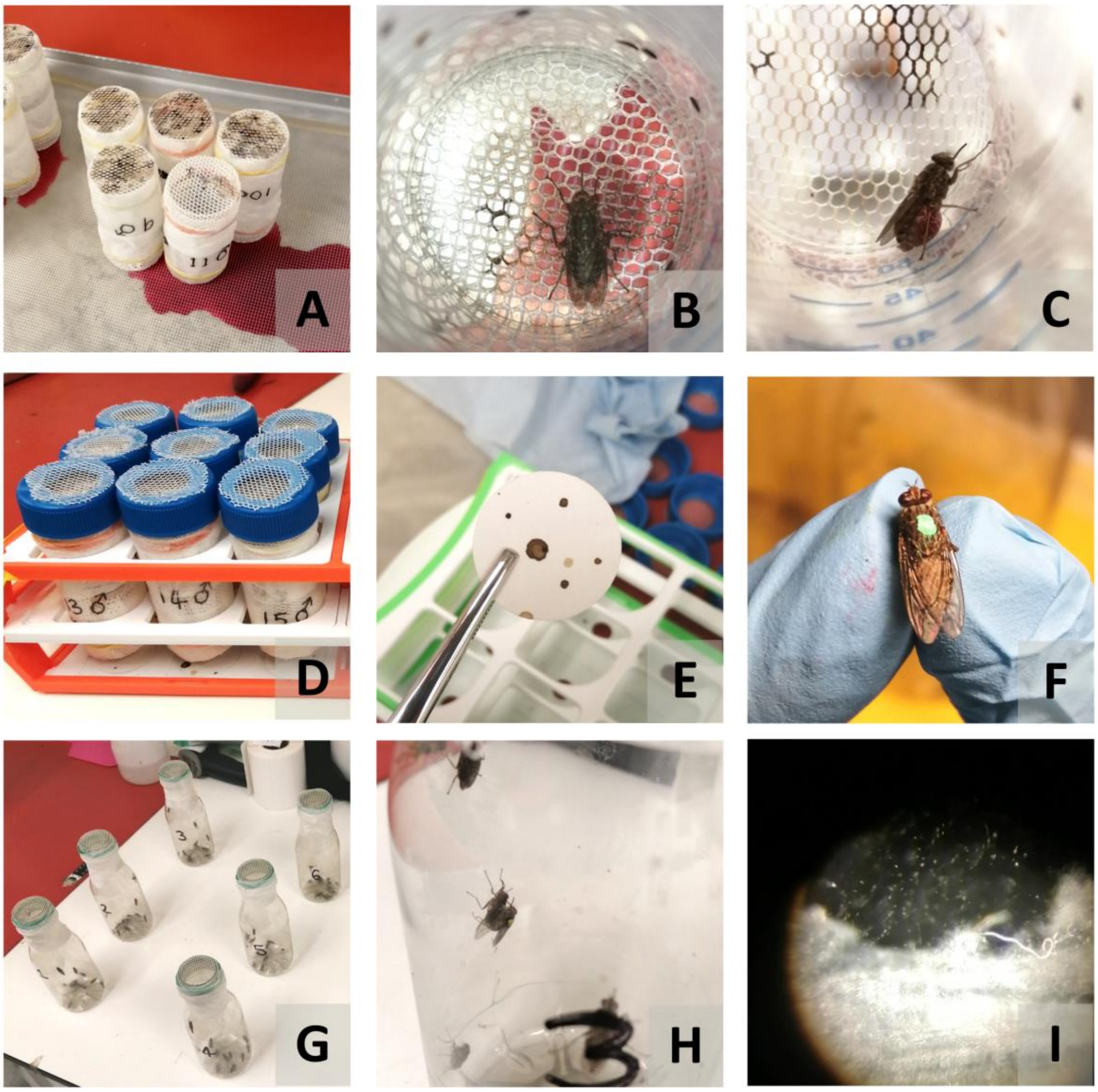

S1 Fig: Images from experiments conducted on insectary-reared tsetse. (A) and (B) show tsetse being blood-fed in solitary cells; (C) tsetse resting after bloodmeal; (D) tsetse solitary cells suspended above filter paper discs in rack; (E) collection of tsetse faecal samples on filter paper; (F) an infected tsetse marked with green oil paint; (G) experiment trap cages; (H) two infected tsetse (fly IDs 87 and 109) copulating inside trap cage during experiment; (I) dissected tsetse midgut infected with *T. brucei* as viewed under a microscope (400X).
