# Supplementary material for: Caught in a trap: DNA contamination in tsetse xenomonitoring can lead to over-estimates of Trypanosoma brucei infection
Source: PLoS Negl Trop Dis. 2024 Aug 12;18(8):e0012095. doi: 10.1371/journal.pntd.0012095 (PMC11341098; doi:10.1371/journal.pntd.0012095)
Supplement: S2 Fig — Red arrow indicates target 173bp TBR product. NEC = negative extraction control. LAD = 100bp ladder. (PDF) [file pntd.0012095.s004.pdf]

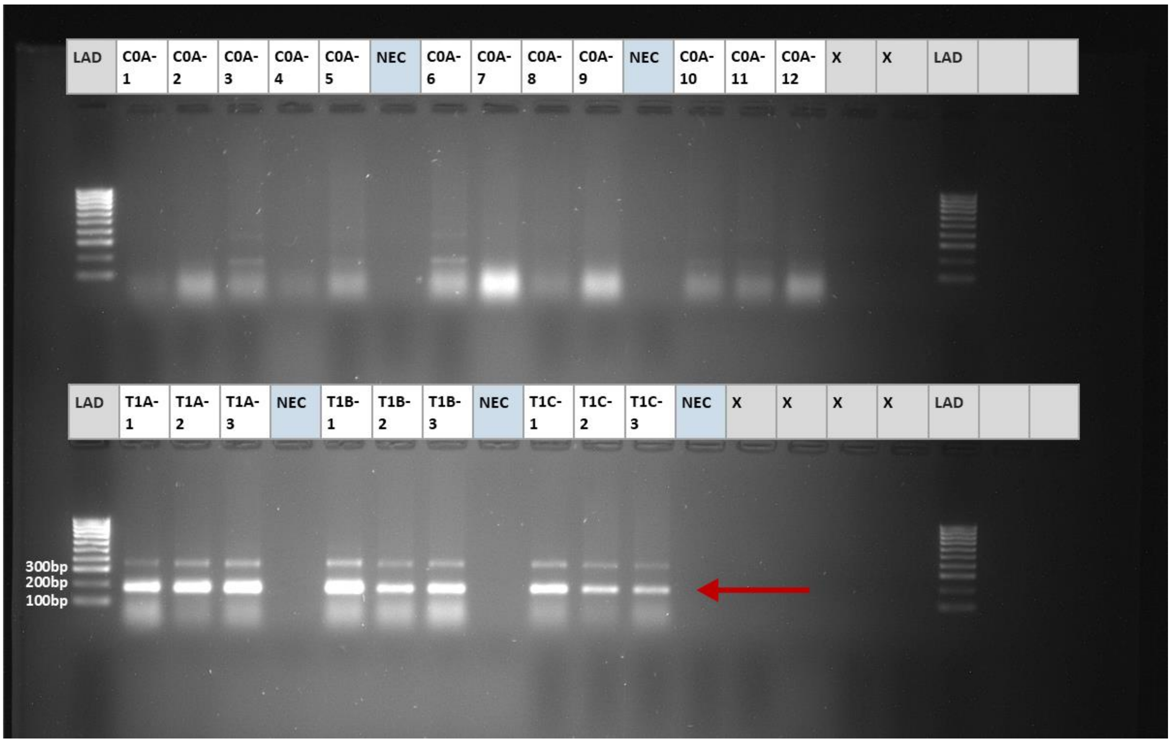

S2 Fig: Gel electrophoresis image from TBR-PCR screening of UFs in C0-A control trap (top row) and naïve flies in T1 control traps (bottom row). Red arrow indicates target 173bp TBR product. NEC = negative extraction control. LAD = 100bp ladder.
