# Supplementary material for: Caught in a trap: DNA contamination in tsetse xenomonitoring can lead to over-estimates of Trypanosoma brucei infection
Source: PLoS Negl Trop Dis. 2024 Aug 12;18(8):e0012095. doi: 10.1371/journal.pntd.0012095 (PMC11341098; doi:10.1371/journal.pntd.0012095)
Supplement: S3 Fig — S3A: A box-and-whisker plot (left axis) showing Cq values obtained from TBR-qPCR screening of faecal samples at four timepoints and eventual whole fly DNA from a subset of infected (IF) and refractory uninfected flies (UF) that were subject to dissection ante-mortem (n = 44). Samples from infected flies are in red, samples from refractory (uninfected) flies are in blue. The bars (right axis) shows the proportion of faecal samples recording TBR-qPCR amplification (where samples were available). The crosses represent the mean Cq values. The amount of T. brucei DNA detected in IF samples was consistently higher than that detected in UFs. Where amplification was recorded, there was a significant difference between mean TBR-qPCR Cq values from infected (mean = 17.57) and uninfected whole flies at 20 days (mean = 33.54, p = <0.0001). The midgut infection rate of this subset was 57% (25/44). S3B: Diagnostic positive predictive value (PPV) and negative predictive value (NPV) calculations for TBR-qPCR screening of tsetse faecal samples as a diagnosis of infection. Faecal samples collected 10–14 days post-inoculation that tested positive (TBR-qPCR) were highly likely to originate from an infected fly, with diagnostic positive predictive value (PPV) of 91% and negative predictive value (NPV) of 85% A positive TBR-qPCR result (‘qPCR_Y’) was any sample recording amplification (Cq < 40). A negative TBR-qPCR result (‘TBR-qPCR_N’) was any sample that did not record amplification. Infected (‘Infected_Y’) was any fly confirmed as having mature midgut infection by microscopy, whilst uninfected (‘Infected_N’) was any fly confirmed as having no visible trypanosome infection by microscopy. Calculations are based on samples collected 10–12 days post-inoculation and/or 13–14 days post-inoculation. (PDF) [file pntd.0012095.s005.pdf]

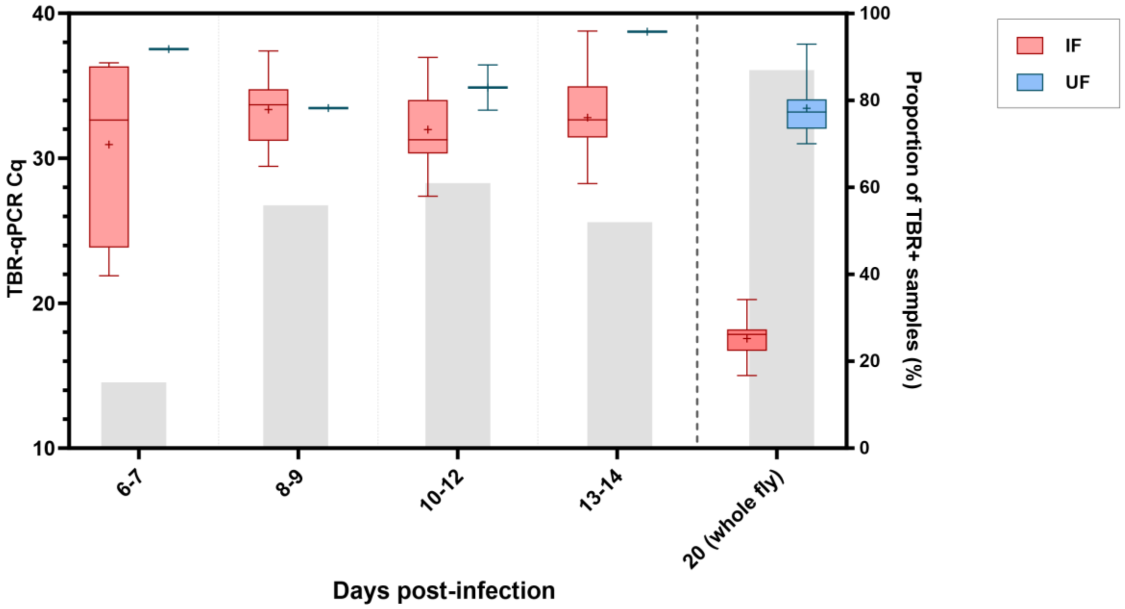

S3 Fig A: A box-and-whisker plot (left axis) showing Cq values obtained from TBR-qPCR screening of faecal samples at four timepoints and eventual whole fly DNA from a subset of infected (IF) and refractory uninfected flies (UF) that were subject to dissection ante-mortem (n=44). Samples from infected flies are in red, samples from refractory (uninfected) flies are in blue. The bars (right axis) shows the proportion of faecal samples recording TBR-qPCR amplification (where samples were available). The crosses represent the mean Cq values. The amount of *T. brucei* DNA detected in IF samples was consistently higher than that detected in UFs. Where amplification was recorded, there was a significant difference between mean TBR-qPCR Cq values from infected (mean=17.57) and uninfected whole flies at 20 days (mean=33.54, *p*=<0.0001). The midgut infection rate of this subset was 57% (25/44).

	Infected_Y	Infected_N		
**qPCR_Y**	40	4	**91%**	*PPV*
**qPCR_N**	5	29	**85%**	*NPV*
	**89%**	**88%**		
	Sensitivity	*Specificity*		

S3 Fig B: Diagnostic positive predictive value (PPV) and negative predictive value (NPV) calculations for TBR-qPCR screening of tsetse faecal samples as a diagnosis of infection. Faecal samples collected 10-14 days post-inoculation that tested positive (TBR-qPCR) were highly likely to originate from an infected fly, with diagnostic positive predictive value (PPV) of 91% and negative predictive value (NPV) of 85% A positive TBR-qPCR result ('qPCR_Y') was any sample recording amplification (Cq < 40). A negative TBR-qPCR result ('TBR-qPCR_N') was any sample that did not record amplification. Infected ('Infected_Y') was any fly confirmed as having mature midgut infection by microscopy, whilst uninfected ('Infected_N') was any fly confirmed as having no visible trypanosome infection by microscopy. Calculations are based on samples collected 10-12 days post-inoculation and/or 13-14 days post-inoculation.
