# Supplementary material for: Caught in a trap: DNA contamination in tsetse xenomonitoring can lead to over-estimates of Trypanosoma brucei infection
Source: PLoS Negl Trop Dis. 2024 Aug 12;18(8):e0012095. doi: 10.1371/journal.pntd.0012095 (PMC11341098; doi:10.1371/journal.pntd.0012095)
Supplement: S4 Fig — The left Y axis displays individual fly TBR-qPCR Cq values, plotted as black, circular symbols. The right Y axis displays number of flies caught in each catch, displayed as a stacked bar chart. Red shows the number of flies testing TBR-positive, blue shows the number of flies testing TBR negative, and grey shows the number of flies that were discarded and not collected. *Transect BB is not featured, as it consisted of 1 TBR-negative fly caught in 1 trap (BB17_15). (PDF) [file pntd.0012095.s006.pdf]

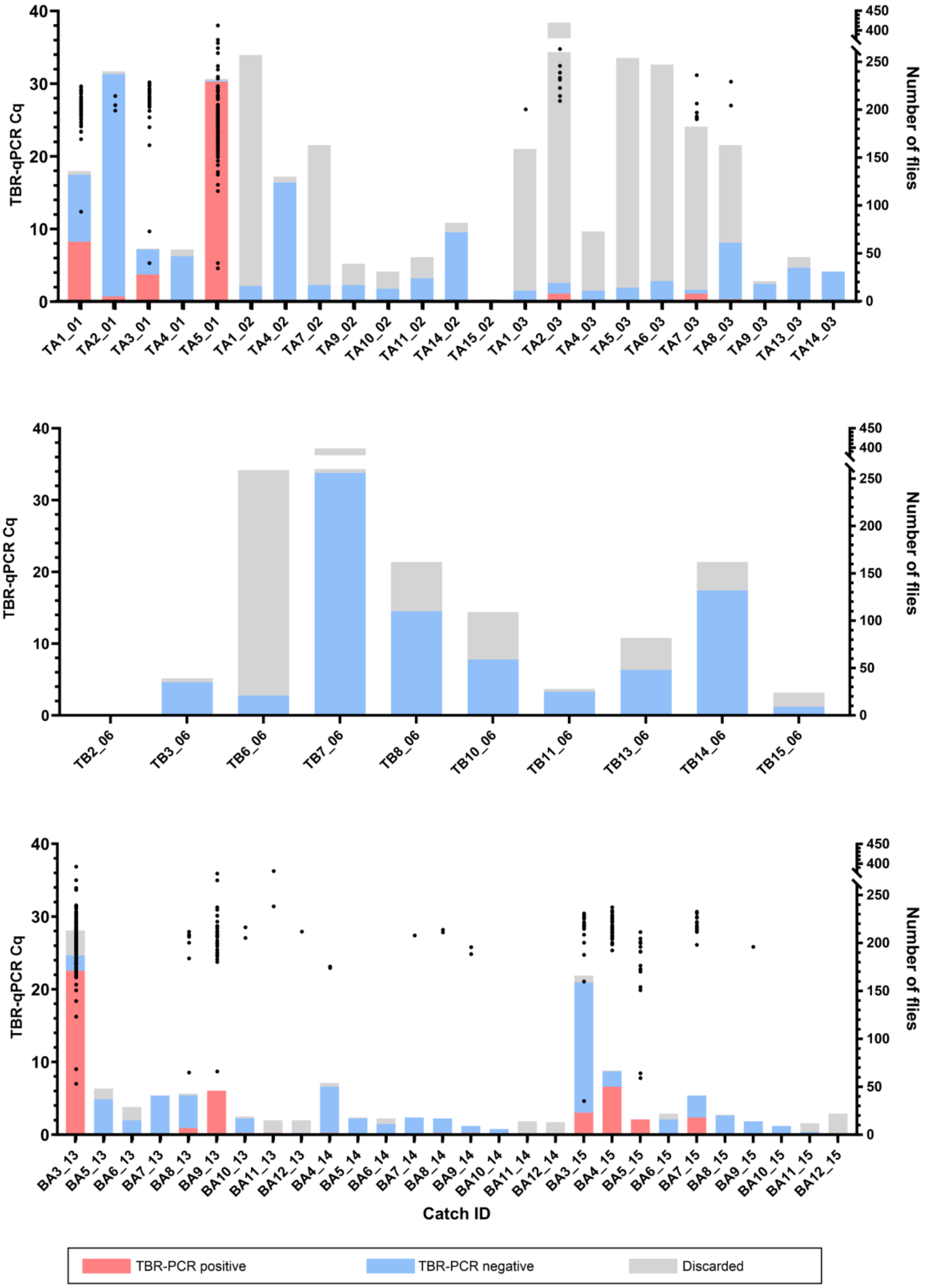

S4 Fig: Plots displaying total catch counts and respective sample TBR-qPCR Cq values for transects TA, TB and BA*. The left Y axis displays individual fly TBR-qPCR Cq values, plotted as black, circular symbols. The right Y axis displays number of flies caught in each catch, displayed as a stacked bar chart. Red shows the number of flies testing TBR-positive, blue shows the number of flies testing TBR negative, and grey shows the number of flies that were discarded and not collected. *Transect BB is not featured, as it consisted of 1 TBR-negative fly caught in 1 trap (BB17_15).
