# Supplementary material for: Caught in a trap: DNA contamination in tsetse xenomonitoring can lead to over-estimates of Trypanosoma brucei infection
Source: PLoS Negl Trop Dis. 2024 Aug 12;18(8):e0012095. doi: 10.1371/journal.pntd.0012095 (PMC11341098; doi:10.1371/journal.pntd.0012095)
Supplement: Attachment — Submitted filename: Caught in a trap_DNA contamination in tsetse xenomonitoring.docx [file pntd.0012095.s008.docx]

**Caught in a trap: DNA contamination in tsetse xenomonitoring can lead to over-estimates of *Trypanosoma* *brucei* infection**

The authors conducted an important study detecting possible contamination and overestimation of trypanosome prevalence in vector flies. The study also demonstrated the sensitivity of the qPCR assays conducted however, the authors conducted TBR-PCR sequencing, and none of their sequences were deposited to NCBI GenBank for accessioning

The manuscript is novel and relevant to the journal as it highlights some of the errors that have been conducted in prevalence studies involving live flies as a means to detect parasitic infection stages within biological vectors

It is recommended that the manuscript be accepted with minor revisions. The authors are encouraged to avoid starting sentences with abbreviations or numbers. Additionally, the authors are advised to deposit and submit all sequences generated.
