# Supplementary material for: Caught in a trap: DNA contamination in tsetse xenomonitoring can lead to over-estimates of Trypanosoma brucei infection
Source: PLoS Negl Trop Dis. 2024 Aug 12;18(8):e0012095. doi: 10.1371/journal.pntd.0012095 (PMC11341098; doi:10.1371/journal.pntd.0012095)
Supplement: Attachment — Submitted filename: Response_to_reviewers_ISALDANHA.docx [file pntd.0012095.s009.docx]

**Point-by-point response to reviewers**

**‘Caught in a trap: DNA contamination in tsetse xenomonitoring can lead to over-estimates of *Trypanosoma brucei* infection’**

All changes made to manuscript are visible under ‘track changes’ in the track changes document. The line numbers used by the authors refer to the line numbers in the ‘track changes’ document.

As similar comments and requests were made by several reviewers across the manuscript, comments have been grouped and a response made to these.

| **Reviewer** | **Comments** | **Response** |
| --- | --- | --- |
| Editors | *The inclusion of a cleaning step to remove external contamination prior to processing for PCR, would have added value to understand the steps necessary to obtain really clean flies; for example, a washing step in water and bleach solution, or even a collection of flies directly into an Ethanol-based solution inside the trap bottles, could reduce/remove cross-contamination. I recommend that the authors include a statement explaining the possible inclusion of this step, but that their goal was to prove cross-contamination initially and that these cleansing steps could be investigated in future.* | We agree that a washing step prior to DNA extraction could reduce or even resolve the environmental contamination. However, the purpose of this study was to determine, in the first instance, whether a viable DNA contamination route existed rather than to explore methods to prevent or mitigate the contamination. Across the literature, there is no evidence of sample-washing being tested or used in the molecular xenomonitoring of tsetse. We also reference several studies with suspiciously high infection rates and/or prevalences, suggestive of contamination.  Nonetheless, it is possible that changes to trap cage could decrease or prevent contamination (see Discussion, line 632 onwards). Testing these additional hypotheses was beyond the scope of the current study but should be the focus of future research.  To address this within the manuscript, we have expanded part of the Discussion (lines 641-657) to include sample-washing as a potential avenue for future research, with references to papers that have used bleach to remove environmental contaminant DNA from arthropod samples. A sentence has also been added to clarify the purpose of the study (line 655-656), and the final concluding sentence of the paragraph has been amended to highlight sample-washing as an area for future research (line 656-657)  The quantitative methods described in this study are most suitable for samples which have already been collected and suspected (or known) to have been affected by the DNA contamination described in this study. We acknowledge that quantitative PCR is not suitable for all settings, and agree that preventative measures are the priority and should be explored. However, for instances where sample-washing or other contamination prevention measures are not feasible, quantitative PCR provides a viable method for calculating more accurate infection prevalences. To clarify this, a sentence in the hypothesis has been amended to reflect the intended usage of the quantitative method (line 176). |
| Reviewer 2 | (Methods) *However, my major criticism is that the authors do not report on the possibility of washing/cleaning the flies before molecular processing to limit these contaminations, it may be an easier way without the need for the whole battery of molecular test that may not very operational for large scale surveillance systems. More precisely: 134-136 given this risk of contamination why not wash the different flies before qPCR to prevent the detection of environmental contamination? Similarly, the quantitative methods displayed lines 447-452 are very good but again what about washing the samples before further processing is the contamination is only external.* |  |
| Reviewer 3 | (Methods) *The major shortcoming of this work is that they have excluded a clean-up stage in sample processing using established approaches (bleach and wash). This would have provided valuable information on whether end-point PCR is still valid as a protocol for xenomonitoring. The proposed qPCR is good, but not readily available in labs, especially where tropical infectious diseases are found.* |  |
| Reviewer 2 | (Conclusions) *No, the key point missing is the preprocessing of samples that could sort of void the need for the manuscript or instead increase the importance of it.* |  |
| Reviewer 3 | (Conclusions) *This is an interesting study, because it can help to clarify shortcomings of xenomonitoring, and to provide opportunities to improve the approach. The very basic step of cleaning the surface of whole insects should be included.* |  |
| Editors | *To deposit sequences generated to NCBI genbank.* | A total of 69 *T. brucei* minisatellite (TBR-PCR product) sequences have been deposited in NCBI Genbank under accession numbers PP942745 to PP942812. The results section (lines 501-503) has been updated to reflect this. |
| Reviewer 1 | (Conclusions) *However, generated sequence data from TBR-PCR are not deposited to NCBI GenBank* |  |
| Editors | *Avoid starting sentences with abbreviations, eg. line 490.* | The full species name (*Trypanosoma brucei*) has been added where the abbreviated form (‘*T. brucei*’) previously started a sentence (lines 317, 367 and 564). |
| Editors | *Line 261: Write in full.*  *Line 304: Write in full.* |  |
| Editors | *Add references for arbovirus examples, Line 93-96.* | The sentence has been amended and a citation has been added to include molecular xenomonitoring of Dengue, Chikungunya and Zika (line 99). |
| Reviewer 2 | *Lines 93-96, it would be good to add arboviruses references/examples.* |  |
| Editors | *Perhaps a better photograph of flies inside the collection bottle (Fig 2).* | We acknowledge that the image in the pdf is poor but the original appears to be adequate. We wonder whether the reviewer downloaded and viewed the .tif file of Figure 2 to view, in full resolution, a tsetse fly within a plastic bottle instead of on vegetation. If the editor and reviewers are still not satisfied, then we will remove panel C from Figure 2. |
| Reviewer 3 | (Results) *Take note however that Figure 2 is poor, especially panel C that is unclear. Is this a fly on vegetation or in a trap?* |  |
| Editors | *Line 68: No citation for paragraph starting from line 69-76. Insert relevant citation.* | A relevant citation (for tsetse being the primary vectors of HAT and AAT) has now been added (line 71). |
| Editors | *Line 83: no italics* | All instances of ‘sp.’ have been reformatted and are no longer in italics (lines 25, 52, 69, 83, 516, 664, Figure 2 caption and Table 2 caption). |
| Editors | *Lines 114-115: Citation needed* | A relevant citation (for DNA contamination in PCR) has now been added (line 121). |
| Editors | *Line 153: Provide a ratio of the sexes in brackets* | The fly sex ratio (80 male, 60 female) has been added to the sentence (line 181). |
| Editors | *Line 164: How many males and how many females survived from 106 individuals?* | The fly sex ratio (50 female, 56 male) has been added to the sentence (line 200). |
| Editors | *Line 174: What was it? Fluorescent dye or paint or what??? Please explain.*  *Line 175-176: Move this to line 174.* | As suggested, the details of the oil paint marker for infected flies have been moved to the first mention of the ‘unique identifier’ (line 210). |
| Editors | *Line 235: Manufacturer details in brackets.* | The manufacturer details (QIAGEN, Hilden, Germany) for the DNA extraction kits and tissue disruptor have been placed in brackets and added at lines 286, 292 and 296. |
| Editors | *Line 278: Quantitative PCR…* | ‘qPCR’ has been changed to quantitative PCR (line 336). |
| Editors | *Line 312: Write in words or rephrase to avoid starting a paragraph with a number.* | This sentence has been rephrased (with the addition of ‘the’) to avoid starting the sentence with a number (line 378). |
| Reviewer 2 | (Methods) *…* *what about other trypanosome parasites and cross contamination in the field with thelleria, babesia or any others from biting flies that may also be trapped and so on.* | Whilst exploring the DNA contamination of other parasite species was beyond the scope of the current study, the authors agree that this would be worth exploring in future studies. The possibility of other trypanosome species being implicated in DNA contamination is mentioned in the Discussion (lines 661-663). |
| Reviewer 3 | (Results) *Faecal samples of infected flies were analysed for the presence of trypanosomes (under Trap experiments). Why was it necessary to dissect the flies thereafter?* | Whilst we had indirect indications as to which flies had established midgut infections, based on TBR-qPCR screening of faecal samples, we wanted to confirm infection status post-mortem by using dissection and microscopy to visualise the parasites. This is explained in the Methods section ‘Tsetse dissection and microscopic analysis’. |
| Reviewer 2 | (Conclusions) *The link between the lab and field part should also be made clearer.* | In the final paragraph of the introduction, we have added the following section (lines 173-176):  “To test this hypothesis, we conducted laboratory-based studies to assess whether mixing infected and uninfected tsetse within a cage resulted in both groups of flies being positive for TBR-PCR. We also examined wild-caught tsetse to assess whether there was evidence of cross-contamination occurring in practice. Finally, we developed a means of estimating infection prevalence accurately in settings where contamination is suspected or known to have occurred.” |
